# Cytotoxic Effects during Knock Out of Multiple Porcine Endogenous Retrovirus (PERV) Sequences in the Pig Genome by Zinc Finger Nucleases (ZFN)

**DOI:** 10.1371/journal.pone.0122059

**Published:** 2015-04-24

**Authors:** Marwan Semaan, Daniel Ivanusic, Joachim Denner

**Affiliations:** 1 Robert Koch Institute, Nordufer 20, Berlin, Germany; 2 Freie Universität Berlin, Kaiserswerther Str. 16–18, Berlin, Germany

## Abstract

Xenotransplantation has been proposed as a solution to the shortage of suitable human donors for transplantation and pigs are currently favoured as donor animals. However, xenotransplantation may be associated with the transmission of zoonotic microorganisms. Whereas most porcine microorganisms representing a risk for the human recipient may be eliminated by designated pathogen free breeding, multiple copies of porcine endogenous retroviruses (PERVs) are integrated in the genome of all pigs and cannot be eliminated this way. PERVs are released as infectious particles and infect human cells. The zinc finger nuclease (ZFN) technology allows knocking out specifically cellular genes, however it was not yet used to eliminate multiple integrated proviral sequences with a strong conservation in the target sequence. To reduce the risk of horizontal PERV transmission and to knock out as many as possible proviruses, for the first time the powerful tool of the ZFN technology was used. ZFN were designed to bind specifically to sequences conserved in all known replication-competent proviruses. Expression and transport of the ZFN into the nucleus was shown by Western blot analysis, co-localisation analysis, PLA and FRET. Survival of transfected cells was analysed using fluorescent ZFN and cell counting. After transfection a strong expression of the ZFN proteins and a co-localisation of the expressed ZFN proteins were shown. However, expression of the ZFN was found to be extremely toxic for the transfected cells. The induced cytotoxicity was likely due to the specific cutting of the high copy number of the PERV proviruses, which is also commonly observed when ZFN with low specificity cleave numerous off-target sites in a genome. This is the first attempt to knock out multiple, nearly identical, genes in a cellular genome using ZFN. The attempt failed, and other strategies should be used to prevent PERV transmission.

## Introduction

Xenotransplantation using porcine cells, tissues or organs may reduce the widening gap between demand and supply of human donor organs [1]. However, xenotransplantation may be associated with transmission of zoonotic microorganisms [2]. Whereas most of the potential microbes can be eliminated by breeding under designated pathogen free conditions, this is not possible for porcine endogenous retroviruses (PERVs). PERVs belong to the gammaretrovirus family, they are integrated in the genome of all pigs and were shown to infect human cells *in vitro* [3–5]. Three subtypes of PERVs were identified, PERV-A and PERV-B, which are ubiquitous and human tropic, and PERV-C, which is not present in all pigs and infects only pig cells [5]. To date, no *in vivo* transmission of PERVs to humans, primates and small animals following experimental and clinical xenotransplantation using pig cells and tissues (with and without immunosuppression) or inoculation of concentrated virus was observed (for review see [6]). Nevertheless, since many retroviruses are pathogenic and able to induce tumours as well as an immunodeficiency, this cannot be excluded for PERVs, especially because it infects human cells and because pharmaceutical immunosuppression may be an important factor in xenotransplantation. In recent years different strategies were developed to reduce the risk of PERV transmission to the recipient [7]. These strategies included selection of pig strains with a low expression of PERV-A and PERV-B. In addition it was recommended to select pigs lacking PERV-C in the genome in order to prevent recombination between PERV-A and PERV-C. Such PERV-A/C recombinants infect human cells and are characterised by high replication rates in comparison with the parental virus (for review see [8]). Other strategies included generation of transgenic pigs expressing PERV-specific small interfering (si)RNA [9–12] and the induction of neutralising antibodies [13–15].

Recently the zinc finger nuclease (ZFN) technology was developed to generate precisely targeted *in vitro* or *in vivo* genomic edits with targeted gene deletions (knock outs), integrations, or modifications [16]. ZFNs are a class of engineered DNA-binding proteins that facilitate genome editing by creating a double-stranded break in DNA at a user-specified location [17]. A double-stranded break is important for site-specific mutagenesis in that it stimulates the cell’s natural DNA-repair processes, namely homologous recombination and non-homologous end joining [17]. By taking advantage of the errors of this DNA repair machinery, ZFN can be used to precisely alter the genomes of higher organisms [18, 19]. The non-specific cleavage domain from restriction endonuclease FokI is typically used as the cleavage domain and the DNA-binding domains typically contain between three and six individual zinc finger repeats and can each recognize between 9 and 18 base pairs. Many engineered ZFN have been shown to have excellent binding specificity in vitro, whereas others are less specific [20, 21]. ZFN have become useful reagents for manipulating the genomes of many plants and animals [18, 19], including pigs [22–30]. Monoallelic and biallelic knock outs were described, and in most published *in vitro* and *in vivo* experiments single genes were knocked out, only in rare cases two genes [24].

However, PERVs are present in multiple copies (60 to 150 copies) in the genome of pigs [31]. The *pol* gene of all proviruses is highly conserved. Therefore it was of great interest to investigate, whether the ZFN technology may be used to knock out multiple, nearly identical genes, in this case the integrated proviruses of PERV. In order to test this, ZFN were designed corresponding to highly conserved sequences in the pol region and their effect on PERV expression was studied. Using different methods we demonstrated that the ZFN were strongly expressed in the nucleus of PERV-producing pig cells and PERV-infected human cells. However, we also observed, that the transfected cells died after expression of the ZFN in the nucleus, possibly by cutting the numerous PERV proviruses.

## Material and Methods

### Zinc finger nucleases specific for PERV

Zinc finger nucleases targeting the PERV genes were designed, cloned and validated by Sigma-Aldrich, St. Louis, MO, USA. Among 67 ZFN candidates targeting the *pol* gene of PERV, 10 ZFNs were found to target highly conserved regions of the highly conserved *pol* gene of PERV when the sequences of seven of different PERV sequences were aligned (S1 Fig). From these 10 candidates, 3 most active ZFN pairs were selected. The binding sites of the ZFN pairs as well as their relative Mel-1 activity are shown in Table 1. The lower case letters correspond to the cutting site where the double-strand breaks will take place.

**Table 1:** Design of zinc finger nucleases targeting the PERV genes.

ZFN	Target sequence	Position in PERV-A^a^	Relative Mel-1 Activity ^b^
PZFN1/PZFN2 (Set 1)	CGCAAGGACCTTACAgacatACCGCTGACTGGAGAA	3957..3995	116.4%
PZFN3/PZFN4 (Set 2)	AACATCGTTCGGCAGcccccAGACCGATGGATGAC	4405..4439	109.9%
PZFN5/PZFN6 (Set 3)	GGCCCCAACCACAGCCAAacaagtGAGAGAGTTTTTGGG	4558..4592	98.8%

^a^ Accession nr. AJ293656

^b^ Compared with positive control

### Cloning of ZFN1-CFP and ZFN2-YFP

In order to study the expression of ZFN proteins and locate them in the cells, the sequences corresponding to the fluorescent markers cyan fluorescent protein (CFP) and yellow fluorescent protein (YFP) were inserted in the ZFN plasmids at the 3’ side of the ZFN1 and ZFN2 respectively. The CFP sequence was amplified from a pcDNA-CFP (containing the improved CFP designated SCFP3A, [36]) using the primers for TTTTTAGATCTGCCGCCGCCATGGTGAGCAAGGGCGAGGAG and rev TTTTTCTCGAGCGGAACCTTTCCGGACTTGTACAGCT. The PCR product was purified and inserted into the ZFN1 plasmid by digestion ligation using BglII and XhoI. For ZFN2 a mutagenesis PCR was carried out to replace a stop codon by a BamHI restriction site using the primers for CAACGGCGAGATCAACTTCGGATCCCTCGAGTCTAGAGGGCCCG and rev CGGGCCCTCTAGACTCGAGGGATCCGAAGTTGATCTCGCCGTTG. The YFP insert was obtained by digestion of pCMV-CD63-YFP with XhoI/ApaI. The insert was purified by gel extraction using the Invisorb Spin DNA Extraction Kit (Stratec, Berlin, Germany) and then inserted in an XhoI/ApaI digested ZFN2 plasmid. In order to avoid that the fluorescent tags do not interact with each other which may lead to false positive results, fluorescent hybrid protein expression vectors with modified SCFP3A and SYFP2 templates [27] were used to improve the brightness and monomeric properties of the expressed proteins. For simplicity we call them YFP and CFP.

### Cell culture, cell counting and infection of 293 cells

Porcine embryonic kidney PK-15 cells (ATCC CRL-33) and human embryonic kidney 293 cells (ATCC CRL-1573) were cultured in Dulbecco’s modified Eagle medium (DMEM) supplemented with 10% heat-inactivated fetal calf serum (FCS), 100 IU/ml penicillin, 100 μg/ml streptomycin (PAA Laboratories, Cölbe, Germany) and 2 mM L-glutamine (Biochrom AG, Berlin, Germany). Cells were grown in a 6-well plate, and after 24 h PERV-containing cell-free filtered through 0.45 μm filters (Schleicher & Schuell Bioscience, Dassel, Germany) supernatants were then added to the 293 cells and incubated for 3 days. Cells were counted in triplicates using a Neubauer chamber.

### Nucleofection

Cells were transfected using the Amaxa Nucleofector II device together with the cell line nucleofector kit V (Lonza, Basel, Switzerland). Cells were splitted 2 days before nucleofection. Directly before nucleofection cells were trypsinized and washed gently with PBS, 1 x 10^6^ cells were resuspended in 100 μl of the pre-mixed nucleofector solution (82 μl nucleofection buffer and 18 μl supplement solution). DNA plasmids were added and the mixture was transferred into a 2 mm electroporation cuvette and nucleofected with the program A-023 for 293 cells, and T-023 for PK-15 cells. After nucleofection cells were immediately transferred into in a 6-well plate containing 2 ml pre-warmed medium.

### Surveyor nuclease mismatch assay

The ZFN activity was detected using the Transgenomic Surveyor mutation detection kit (Transgenomic, Glasgow, UK) which takes advantage of the non-homologous end joining DNA repair system triggered by the double strand break at the ZFN target site. The Surveyor nuclease is an endonuclease that cleaves both strands of a DNA at sites of base mismatch. In a first step the ZFN target region was amplified by PCR from genomic DNA using the primers (for: CGAAGGCACTACTGCTGGAA, rev: CGTTGGT CATCCATCGGTCT). If the ZFNs were active the PCR resulted in a pool of mutated and unmutated amplicons which were hybridized by heating and slowly cooling to form hetero- and homoduplexes. Heteroduplexes contain a “bubble” formed at the site of mismatch, which will be cut by the Surveyor nuclease. The cleavage products were analysed by agarose gel electrophoresis or polyacrylamide gel electrophoresis.

### Agarose gel electrophoresis

A 2% gel was prepared by dissolving agarose powder in 1x TAE Buffer (50x: 50 mM EDTA, 1 M acetic acid, pH 8.0, Carl Roth, Karlsruhe, Germany) and microwave heating for about 1 minute. The solution was then cooled to approximately 55–60°C and ethidium bromide (EtBr) was added (0.1 μl / ml of 10 mg/ml stock solution, Sigma Aldrich, Steinheim Germany), 1x TAE buffer was used for electrophoresis; the DNA samples were diluted into DNA loading-buffer (Fermentas, St. Leon-Rot, Germany), a current between 1–10 Volts/cm was applied. The DNA was visualized by UV using the CHEMOCAM Imager 3.2 (INTAS, Göttingen, Germany) and the size of the DNA was determined using the GeneRuler DNA Ladder Mix (Fermentas, St. Leon-Rot, Germany). In order to analyse the concentration of the amplicons by band intensity, the ImageJ software (NIH, Bethesda, MD, USA) was used.

### Polyacrylamide gels for resolving small DNA fragments

For a better resolution, a 10% polyacrylamide gel was prepared. Gels were poured into a BioRad gel apparatus (BioRad, Munich, Germany). After polymerization gels were left at 4°C overnight before use. Gels were then fixed into a BioRad electrophoresis chamber filled with 1x TBE buffer. DNA samples were mixed with 6x loading buffer (Fermentas, St. Leon-Rot, Germany). Electrophoresis was carried out at constant voltage of 110 V for 70–100 min. Gels were then stained for 15–20 min in 1x TBE containing 0.1 μl / ml of 10 mg/ml stock solution EtBr and visualized by UV using the CHEMOCAM Imager 3.2 (INTAS, Göttingen, Germany).

### *In situ* PLA

PK-15 cells (3 × 10^5^ cells/well) were seeded in 6 wells, after 5 h MetafectenePro (Biontex, Munich, Germany) was used for co-transfection of cells with the vectors pZFN1 and pZFN2 (Sigma Aldrich) and empty FLAG expressing vector pCMV-Tag2B (Stratagene, Heidelberg, Germany) as control. Transfected PK-15 cells were incubated for 24 h and fixed for 20 min in 2% paraformaldehyde dissolved in PBS. After the fixation cells were washed with PBS and detached from 6 wells. The fixed cell suspension was dropped on poly-L-lysine coated slides (Polylysine, Thermo Scientific, Braunschweig, Germany) within a 2 cm^2^ PAP-pen (Sigma Aldrich, Steinheim, Germany) marked circle barrier. Cells were dried on glass slides, washed with PBS and rinsed in 0.4% saponin (Carl Roth, Karlsruhe, Germany) for permeabilisation. After 60 min cells were blocked for 2 h at room temperature in 30% normal donkey serum (Jackson Immuno Research, West Grove, PA, USA). To detect ZFN protein expression we used for PLA goat anti-FLAG (NB600-344, NovusBio, Littleton, CO, USA). The cells are incubated with primary antibodies (5 μg/ml) overnight at 4°C in a humid chamber. The following day, the cells were twice washed briefly with Duolink II buffer A and left for 2 h in Duolink II buffer A. After this extended washing step the cells were blocked again with 30% normal donkey serum for 1 h at room temperature. After the blocking all further procedures were applied accordingly the Duolink II manual using anti-goat PLUS and anti-goat MINUS as PLA probes. Cells were mounted in Duolink II mounting medium (DAPI for nucleus staining). All Duolink II detection reagents, mounting media and PLA probes produced by Olink Bioscience were obtained from Sigma Aldrich, Steinheim, Germany.

### Confocal laser scanning microscopy (cLSM) and image analysis

PK-15 cells (1 x 10^4^/well) were seeded into standard bottom 8 well μ-slides (Ibidi, Munich, Germany) and transiently transfected with the following plasmids: (i) pZFN1-CFP and pZFN2-YFP, (ii) a plasmid pCFP-YFP expressing CFP-YFP protein as positive FRET control, and (iii) two plasmids pCFP/pYFP expressing unfused CFP and YFP. All plasmids were transfected alone or together in order to acquire a triple set of images from the donor, the acceptor and donor/acceptor. We used for each well 0.3 μg plasmid DNA and MetafectenePro (Biontex, Munich, Germany) as transfection reagent according to the manufacturer's instructions. One day after transfection cells were washed twice with PBS, and fixed for 20 minutes using 2% paraformaldehyde dissolved in PBS, washed with PBS and mounted in glycerol containing 0.1% *p*-phenylenediamine (Sigma Aldrich, Steinheim, Germany). All images were acquired using a ZEISS 780 confocal inverted microscope and a 63 x oil immersion objective (Carl Zeiss, Oberkochen, Germany) was used. Images for FRET analysis were obtained using multitrack instrument settings for CFP and FRET channel, excitation for CFP channel at 405 nm and emission peak at 475 nm/27-nm bandwidth, excitation for FRET channel at 405 nm and emission peak at 527/48-nm bandwidth. YFP signals were additionally detected in a single track with an excitation at 514 nm and emission peak at 527 nm/55-nm bandwidth. Donor and acceptor bleed-through coefficients were determined by acquiring images containing only a donor or acceptor, respectively. NFRET values were measured with ZEISS ZEN 2010 software. Images for PLA were acquired by using ZEISS ZEN instrument settings for Texas Red analogue dye and DAPI staining.

### Preparation of cell lysates, nuclear and cytoplasmic protein extracts

To prepare cell lysates, cells were washed three times in PBS thus removing traces of FCS (300 x g, 5 min, RT). Cells were lysed by adding a NP-40 lysis buffer containing 1.0% NP-40 (Sigma Aldrich, Steinheim, Germany), 150 mM NaCl, 50 mM Tris, pH 8.0 (Carl Roth, Karlsruhe, Germany) in a ratio of 1 μl of the buffer per 10^4^ cells per. Cells were then left on ice for 10 min and subsequently centrifuged at 10000–12000 rpm for 10 min at 4°C. The supernatant was recovered, aliquoted and frozen at -80°C until used. To detect the presence of ZFN proteins in the nucleus of the nucleofected cells by Western blots, nuclear and cytoplasmatic lysates were prepared using a NE-PER nuclear protein extraction kit (Thermo Scientific, Ulm, Germany), which enables the stepwise lysis of cells and extraction of the cytoplasmic part keeping the nucleus intact. A second step of nuclear lysis allowed the extraction of nuclear proteins without genomic DNA and mRNA contaminations. Briefly, cells were trypsinized and washed with PBS. Pellets were then resuspended in 100 μl of ice cold ice cytoplasmic extraction reagent I (CERI) by vigorous vortexing for 15 sec. After incubation on ice for 10 min, 5.5 μl of CERII were added and mixed by vortexing for 5 sec and incubated on ice for 1 min. After centrifugation for 5 min at maximum speed (16000 x g) at 4°C the supernatant containing the cytoplasmic extract is transferred to a new tube. The insoluble pellet which contains the nuclei was resuspended in ice-cold nuclear extraction reagent (NER) and incubated on ice for 40 min with vortexing for 15 sec every 10 min. After centrifugation at maximum speed for 10 min the supernatant (nuclear extract) was transferred to a new tube and stored at -80°C until use.

### Western blot analysis

The electrophoretic transfer of proteins from SDS gels to polyvinylidene difluoride (PVDF) 0.2 μm membranes (Millipore, Schwalbach, Germany) was performed as described. The SDS gel was placed for 10 minutes in a transfer buffer. The PVDF membrane was pre-wetted in methanol then soaked for 10 min in transfer buffer. A blot sandwich was prepared by placing one or two pre-wetted blotting paper sheet on the anode of a Trans-Blot SD Semi-Dry Transfer Cell (BioRad, Munich, Germany), followed by the PVDF membrane then the SDS-gel which was covered by a second layer of blotting paper sheet. The cathode was placed on the top and the blot was run at 20 V for 25 min. After the protein transfer the membrane was incubated in the blocking buffer on a shaker for 1 h at RT. The primary antibody was then diluted in blocking buffer and added to the membrane. After 1.5 h incubation at RT, alternatively over night at 4°C, the membrane was washed (5 x 5 min) in the wash buffer and incubated with the horseradish peroxidise-conjugated secondary antibody diluted 1:1000 for 1 h at RT. After five times (5 min each) washing the detection was carried out by the enhanced chemoluminescence (ECL Western Blotting Detection Reagents Kit, Pierce, Rockford, IL, USA) system according to the manufacturer’s protocol. The detection solution was mixed 1:1 and given to the membrane for one minute. The chemiluminescence was detected with the chemocam and the time for development varied depending on the intensity of the chemiluminescence. The M2 antibody against the 3xFLAG (Sigma-Aldrich, Steinheim, Germany) diluted 1:500 was used. A anti–β actin antibody raised in mice (Sigma-Aldrich, Steinheim, Germany) diluted 1:5000 and an antibody against the DEAD-box RNA helicase 3 (DDX3, Cell Signaling Technology, Frankfurt, Germany) diluted 1:1000 were used.

### Surveyor nuclease assay

In order to optimize the assay, 2 x 10^6^ PK-15 cells as well as PERV infected 293 cells were transfected with different amounts of plasmid DNA (100 ng, 500 ng, 1 zμg, 2μg, 7.5 μg or 10μg) and three consecutive nucleofections with 2 days intervals were performed using 100 ng, 500 ng or 1 μg ZFN1/ZFN2. DNA was isolated as described above. The ZFN target sequence was amplified using 4 different primer pairs (PCR1-PCR4) and using 3 different polymerases: Optimase polymerase (Transgenomic, Glasgow, UK), the Phusion Hot Start Flex DNA Polymerase (New England Biolabs, Frankfurt, Germany) and the Expand high Fidelity Plus polymerase (Roche, Mannheim, Germany). In a second step PCR products were heated to 95°C for dehybridisation and then cooled down slowly for re-annealing. At this stage the wild type sequences and mutated sequence if present will re-anneal building “bubbles” corresponding to the DNA mismatches, which can be then cut by the surveyor nuclease. 10 μl of each sample were loaded on a 2% agarose gel and the concentration of the PCR amplicon was quantified by band intensity. 40 ng/μl for all samples were estimated. In the third step rehybridized amplicons were treated with the surveyor nuclease. Different combinations of reaction parameters were tested for optimization: different DNA amounts, the enhancer concentration (1 or 2 μl) the MgCl_2_ concentration (0, 0.5 or 1 μl) the nuclease quantity (0.5, 1 or 2 μl), as well as the nuclease working time (20 min, 40 min, 1h or 2h). After stopping the reaction with the stop solution, samples were run on a 2% agarose gel or 10% polyacrylamide gel. In parallel, a G/C control was performed as described by the manufacturer. It consists of two control plasmids with inserts that differ at a single base pair. Treatment with nuclease led to cleavage of the heteroduplexes into two bands (416 and 217 bps).

## Results

### Design of zinc finger nucleases targeting the PERV genes

**Fig 1:**
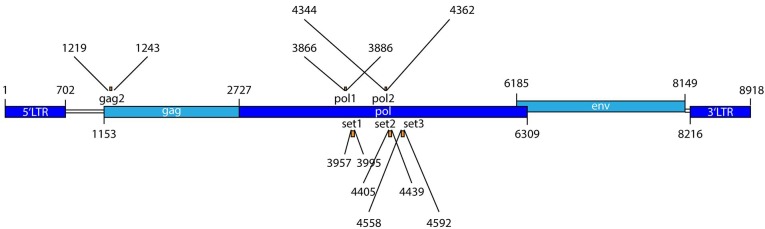
Localisation of the ZFN binding domains (set 1–3) in the sequence of PERV provirus. In addition, sequences of siRNA (gag2, pol1, pol2) successfully used for inhibition of PERV expression were indicated.

Zinc finger nucleases targeting different PERV genes were designed, cloned and validated. Among 67 ZFN candidates targeting the *pol* gene of PERV, 10 ZFNs were found to target highly conserved regions of the pol gene of PERV when the sequences of all known PERV sequences were aligned. These 10 chosen candidates were then tested and the 3 most active ZFN pairs were selected (S1 Fig) and used as plasmid DNA. The binding sites of the ZFN pairs are shown in Table 1. The lower case letters correspond to the cutting site where the double-strand break will take place. To validate the ZFNs, a yeast-based assay was used. In this assay, an artificial ZFN target site is exogenously introduced into yeast cells and the ZFN activity is then measured by using a *MEL1* reporter assay (Table 1). It is important to mention that the binding region of the ZFN is in the same region of *pol* of PERV were two siRNAs, previously shown to effectively knock down PERV expression [9, 11, 12], are binding (Fig 1).

### Detection of ZFN expression by Western blot analysis

**Fig 2:**
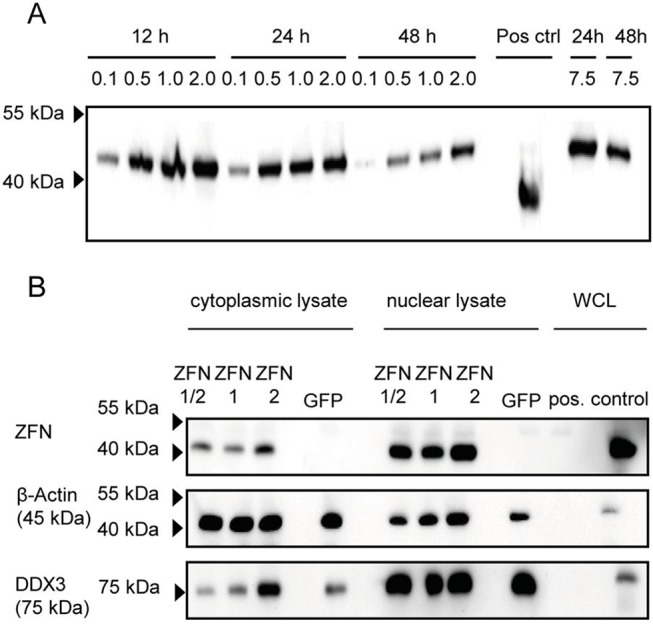
Expression of ZFN proteins in PK-15 cells. **A.** Kinetic of expression of ZFN protein in PK-15 cells after nucleofection of different amounts (0.1 to 7.5 μg) of plasmids corresponding to ZFN set 1 (ZFN1, ZFN2, ZFN1/2). Both expressed proteins carry a 3xFLAG tag and for detection an antibody against the 3xFLAG tag was used. Cell lysate from PK-15 cells expressing an unrelated protein with a 3xFLAF tag and incubated for 2 days after nucleofection was used as positive control (Pos. ctrl). The arrows indicate the marker proteins. **B.** Detection of ZFN proteins in cytoplasmic and nuclear lysates. PK-15 cells were nucleofected with ZFN1 and ZFN2 plasmids together or separately and about 2 x 10^6^ cells per sample were fractionated. Each lane was loaded with nuclear or cytoplasmic extract from about 5 x 10^5^ cells. Whole cell lysate (WCL) from PK-15 cells transfected with pCMV, a vector with a FLAG tag was used as positive control (1 x 10^5^ cells). Anti-Flag antibodies were used for detection of the proteins. The purity of the cytoplasmic and nuclear fractions was analysed using antibodies against β-actin and DDX3, respectively.

In order to analyse the expression of ZFN proteins, 1 x 10^6^ PK-15 cells (expressing and releasing PERV) per sample were nucleofected with plasmids of the ZFN set 1 (0.1 μg, 0.5 μg, 1 μg, 2 μg or 7.5 μg each) and cells were harvested after 12h, 24h and 48h. In a Western blot analysis, ZFN proteins, which were tagged with an N-terminal 3xFLAG, were found expressed at the protein level with a peak at 12 h (Fig 2), at 48 hrs the protein expression was reduced. ZFN proteins contain a nuclear localization signal (NLS), which allows the transportation of ZFN proteins to the nucleus where they can bind to the target DNA. In order to demonstrate the nuclear localisation of the left and the right ZFN proteins, cells were nucleofected with both ZFN1 and ZFN2 plasmids of set 1, either together or separately. After 48h incubation, cells were harvested and nuclear and cytoplasmic lysates were prepared using the Pierce NE-PER nuclear protein extraction kit. In the Western blot analysis bands corresponding to the ZFN proteins with a molecular weight of approximately 50kDa were detected by anti-FLAG antibodies in the cytoplasmic extract but much stronger in the nuclear lysate (Fig 2). To analyse the purity of the cytoplasmic and nuclear samples, antibodies against β-actin and against the DEAD-box RNA helicase 3 (DDX3), typical cytoplasmic and nuclear markers, respectively [32, 33], were used. A negligible contamination of the cytoplasmic extract by the nuclear proteins was detected; in the case of the lysate of cells transfected with ZFN2 alone, this contamination was stronger. Conversely, tracks of β-actin were also detected in nuclear lysates, however it is unclear whether this is a contamination since it was shown that actin plays a physiological role in the nucleus [34, 35].

### Detection of ZFN1/ZFN2 interaction by FRET analysis

**Fig 3:**
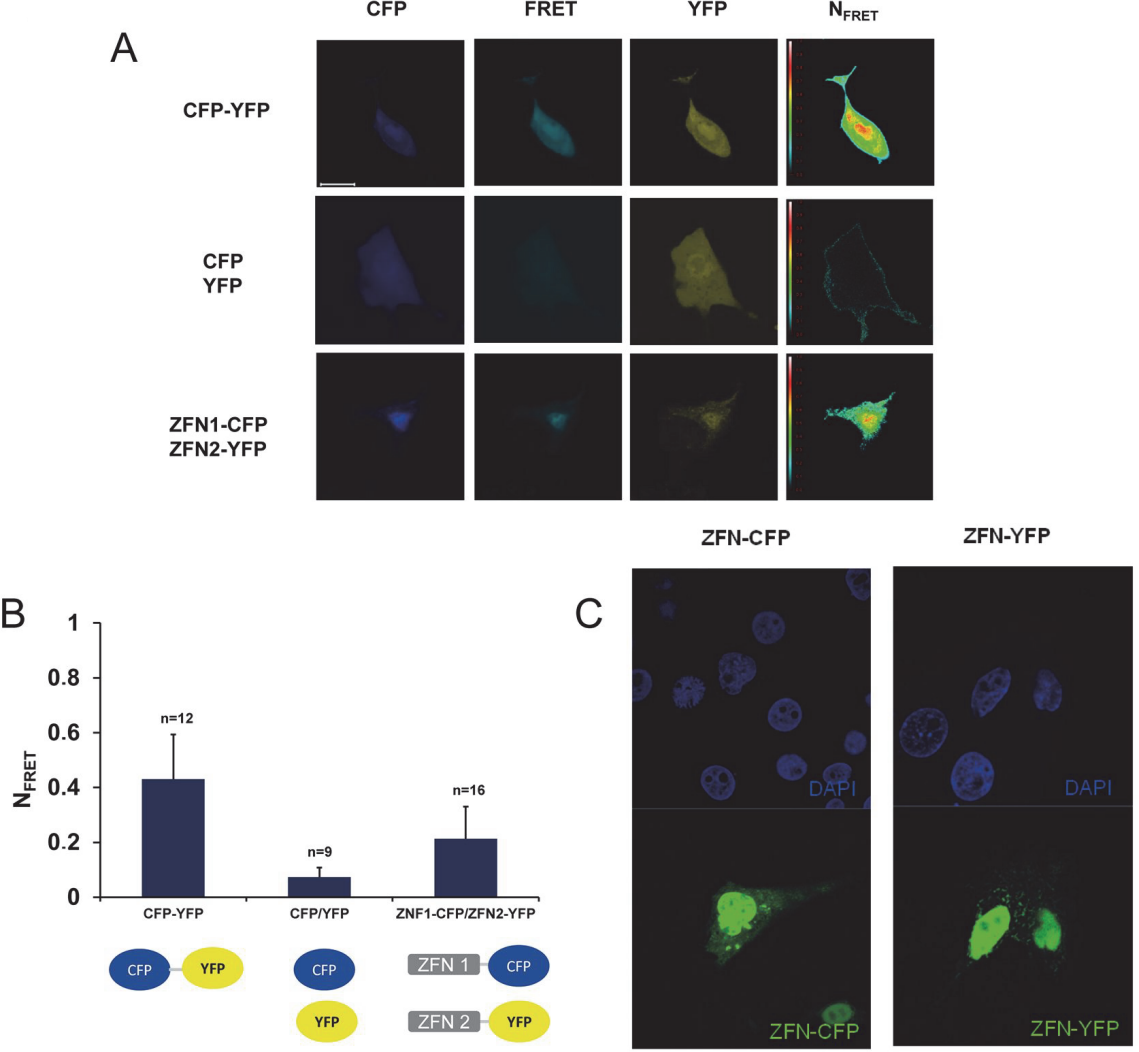
Interaction of ZFN1-CFP and ZFN2-YFP as shown by FRET confocal imaging. **A.** FRET images. 293 cells were transfected with the vector pECFP-EYFP expressing CFP-YFP (row 1), pcDNA-CFP/pCMV-YFP expressing CFP and YFP (row 2) or pZFN1-CFP/pZFN2-YFP expressing ZFN1-CFP and ZFN2-YFP (row 3), fixed 24 h after transfection and then imaged in the following channels: Donor CFP (first column), FRET (second column) and acceptor YFP (third column). The last column displays a corrected and normalized FRET image. NFRET was calculated from the first 3 channels as described in the methods section. Scale bars, 20 μm. NFRET color lookup bar values range from black (0) to red (1). **B.** NFRET intensities of 9–16 cells were measured and the mean NFRET values ±SD are represented. **C.** Expression and localization of the separately transfected ZFN1 fused to CFP or ZFN2 fused to YFP, respectively.

In order to further investigate the expression of ZFN proteins and their localisation in the transfected cells, fluorescence (Förster) resonance energy transfer (FRET) imaging was used. PK-15 cells were transfected with plasmids encoding the cyan fluorescent protein (CFP) and the yellow fluorescent protein (YFP) fused to ZFN1 and ZFN2, respectively, as well as with plasmids coding for YFP fused to CFP or single plasmids coding only for CFP or YFP as controls. To analyse the interactions of the fused ZFN and their localisation, the cells were fixed 24 h after transfection and FRET analysis was performed. The normalized FRET values (NFRET) were obtained from a set of three images (donor, acceptor and co-transfected donor and acceptor). Cells transfected with ZFN1-CFP and ZFN2-YFP revealed high NFRET values (Fig 3). These values are in the same range as the values for expressed YFP fused to CFP, indicating close proximity of both ZFN in form of a heterodimeric pair. In addition, in cells transfected simultaneously with ZFN1-CFP and ZFN2-YFP the expressed proteins were localized predominantly in the nucleus. This and the high NFRET values indicated that the ZFNs were transported to the nucleus and were found in close proximity (less than 10 nm), suggesting that ZFN1-CFP and ZFN2-YFP were interacting. We can exclude that the fluorescent fusion parts of the ZFN, e.g., CFP and YFP, were interacting in an unspecific manner, because in order to generate the plasmids pZFN1-CFP and pZFN2-YFP modified SCFP3A and SYFP2 templates were used to improve the brightness of the fluorescent proteins and their monomeric properties [36]. Both fluorescently tagged ZFN proteins strongly co-localize with DAPI staining of the nucleus (Fig 3).

### Detection of ZFN1 and ZFN2 using the proximity ligation assay (PLA)

**Fig 4:**
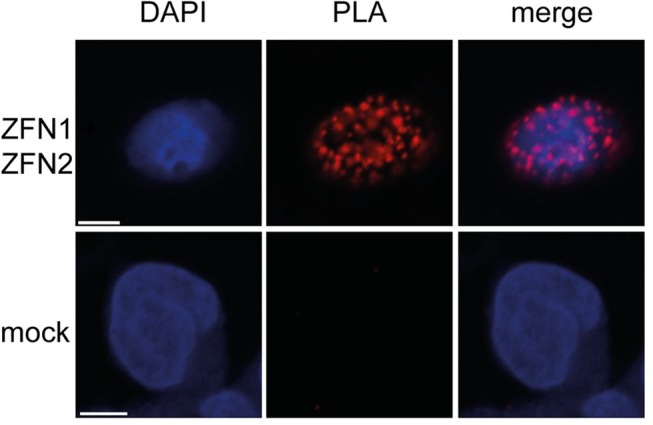
Expression of the ZFN as detected by PLA. PK-15 cells were transfected with the pZFN1 or pZFN2 vectors expressing the 3xFLAG tagged fluorescent proteins ZFN1-CFP or ZFN2-YFP, the nucleus was stained by DAPI and a PLA against the FLAG epitope was performed demonstrating the specific localization of the ZFN in the nucleus. Scale bars, 5 μm.

In order to analyze ZFN1 and ZFN2 without fused CFP and YFP fluorescent tags the unmodified ZFN pair was transiently transfected in PK-15 cells. The advantage of PLA detection is the high specificity and sensitivity for protein epitope recognition. In general, only when two PLA probes (anti-goat PLUS and MINUS) bind to the primary antibody then this results in a PLA signal. The signal from a single protein is several hundredfold increased by the rolling circle amplification during PLA in contrast to classical indirect immunostaining technique [37, 38]. Here, a single protein recognition PLA with goat anti-Flag antibody was performed since both proteins carry the same FLAG epitope. Numerous detectable PLA signals in the nucleus as shown by DAPI staining were found, indicating a specific transport of ZFN1 and ZFN2 into the nucleus (Fig 4). The signals strongly co-localize with the DAPI signal, indicating that the ZFN proteins are in close proximity with the cellular DNA.

### Influence of the ZFN on PERV expression

In order to analyze the influence of the above documented expression of the ZFN1 and ZFN2 on PERV expression, 2 and 5 μg ZFN1 and ZFN2 were transfected into PK-15 or 293 cells infected with supernatant of PERV-producing PK-15 cells or uninfected control cells and incubated for 2 to 5 days. Since the protein expression in PK-15 cells is very low and cannot be quantified, the influence on expression was measured at the RNA level. RNA was isolated on day 2 and day 5 after transfection and using a real-time PCR, expression of PERV and porcine cyclophilin in the case of PK-15 and human GAPDH in the case of PERV-infected 293 cells were measured. No differences in the PERV expression in comparison with the negative control and no changes in PERV expression over time were observed (S2 Fig). As we found out later, we measured PERV expression in surviving cells, cells expressing the ZFN were dead at that time (see below).

### Toxicity of ZFN

In order to analyse whether the ZFN were not functioning or whether other reasons were responsible for this negative result, plasmids expressing ZFN fused to CFP and YFP were used to assess the viability of the transfected cells and the toxicity of ZFN plasmids. For this purpose, PK-15 cells were transiently transfected with 2.5 μg or 7.5 μg ZFN1-CFP and ZFN2-YFP together or separately. Unfused CFP and YFP were used as controls. In the case of single plasmid transfection, a double amount of DNA was transfected. Cells were observed daily using a fluorescence microscope and images were taken on days 1 and 5 after nucleofection (S3 Fig). Fluorescence microscopy revealed that in the case cells expressing ZFN1-YFP and ZFN2-YFP, the fluorescent cells were disappearing progressively until day 5 after transfection, while control cells as well as cells transfected with ZFN1-YFP or ZFN2-YFP separately still showed fluorescence 5 days post-nucleofection (S3 Fig), indicating that the cells expressing ZFN1-YFP and ZFN2-YFP together were dying.

**Fig 5:**
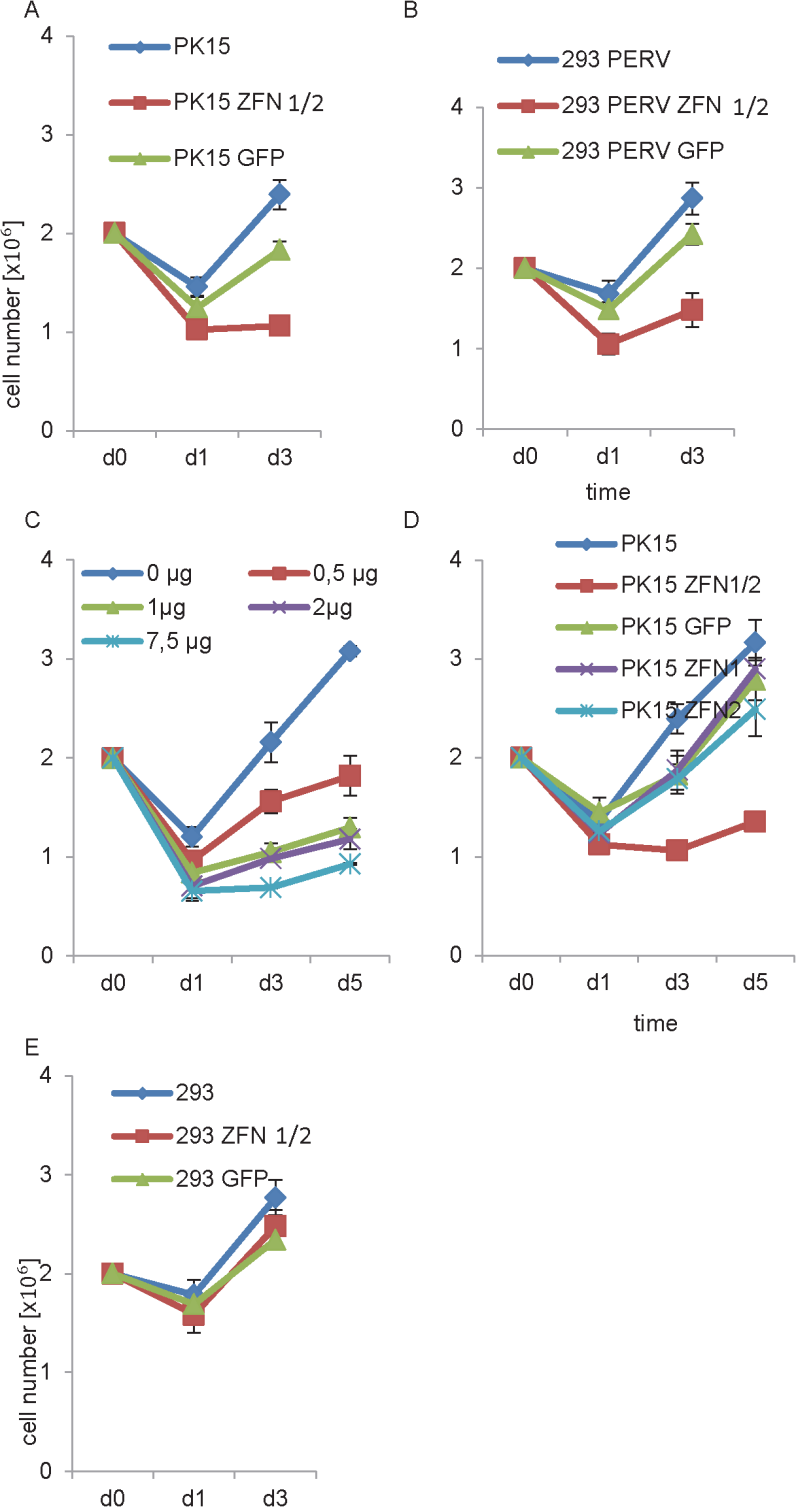
Cell viability after nucleofection with ZFN plasmids. PK-15, PERV-infected and uninfected 293 cells were nucleofected and at days 1, 3 and (in two experiments also 5) post nucleofection the cell number in the cultures was determined. **A.** PK-15 cells were nucleofected with both ZFN, ZFN1 and ZFN2, or with the control plasmid pLVTHM-GFP encoding GFP, or were left untreated. **B.** PERV-infected 293 cells were nucleofected with both ZFN, ZFN1 and ZFN2, or with GFP or were left untreated. **C.** PK-15 cells were left untreated or were nucleofected with different amounts of plasmids expressing ZFN1 and ZFN2 at day 0 and the cell number increased in dependence on the amount of plasmids. **D.** PK-15 cells were left untreated or were nucleofected with pLVTHM-GFP expressing GFP, with ZFN1 alone or ZFN2 alone and both ZFNs together. A double amount of DNA was used in case of transfection with a single plasmid. **E.** Uninfected 293 cells were nucleofected with pLVTHM-GFP (4μg) or ZFN1/ZFN2 (2 μg each) or were left untreated.

Since the linkage of CFP and YFP to the C-terminus of the ZFN proteins near the active centre of the FokI nuclease may interfere with the activity of the ZFN, another toxicity test was performed using the original, unmodified ZFN1 and ZFN2, this means they were not fused to the fluorescent YFP. PK-15 and PERV-infected 293 cells were transfected by nucleofection with bothZFN plasmids, ZFN1 and ZFN2. Although in all cultures a decrease of the cell number was observed on day 1, in cultures with untreated cells or with cells transfected with a control plasmid, GFP, the cell number increased steadily until day 3 (Fig 5A and 5B). However, in cultures with cells treated with ZFN1 and ZFN2 the number of cells was not increasing. Adding different amounts of ZFN 1 and ZFN2 plasmids to PK-15 cells showed that the toxic effect was dose-dependent (Fig 5C). When PK-15 cells were transfected with ZFN1 alone or with ZFN2 alone and with the control GFP vector, no decrease on the cell number compared with untreated PK-15 cells was observed (Fig 5D). Most importantly, when uninfected 293 cells (without integrated PERV proviruses) were transfected with ZFN1 and ZFN2, no effect on the cell number was observed (Fig 5E). This control clearly demonstrates that ZNF1 and ZNF2 are not per se cytotoxic in uninfected 293 cells, but only in the context of PERV.

### Functional analysis of the ZFN

**Fig 6:**
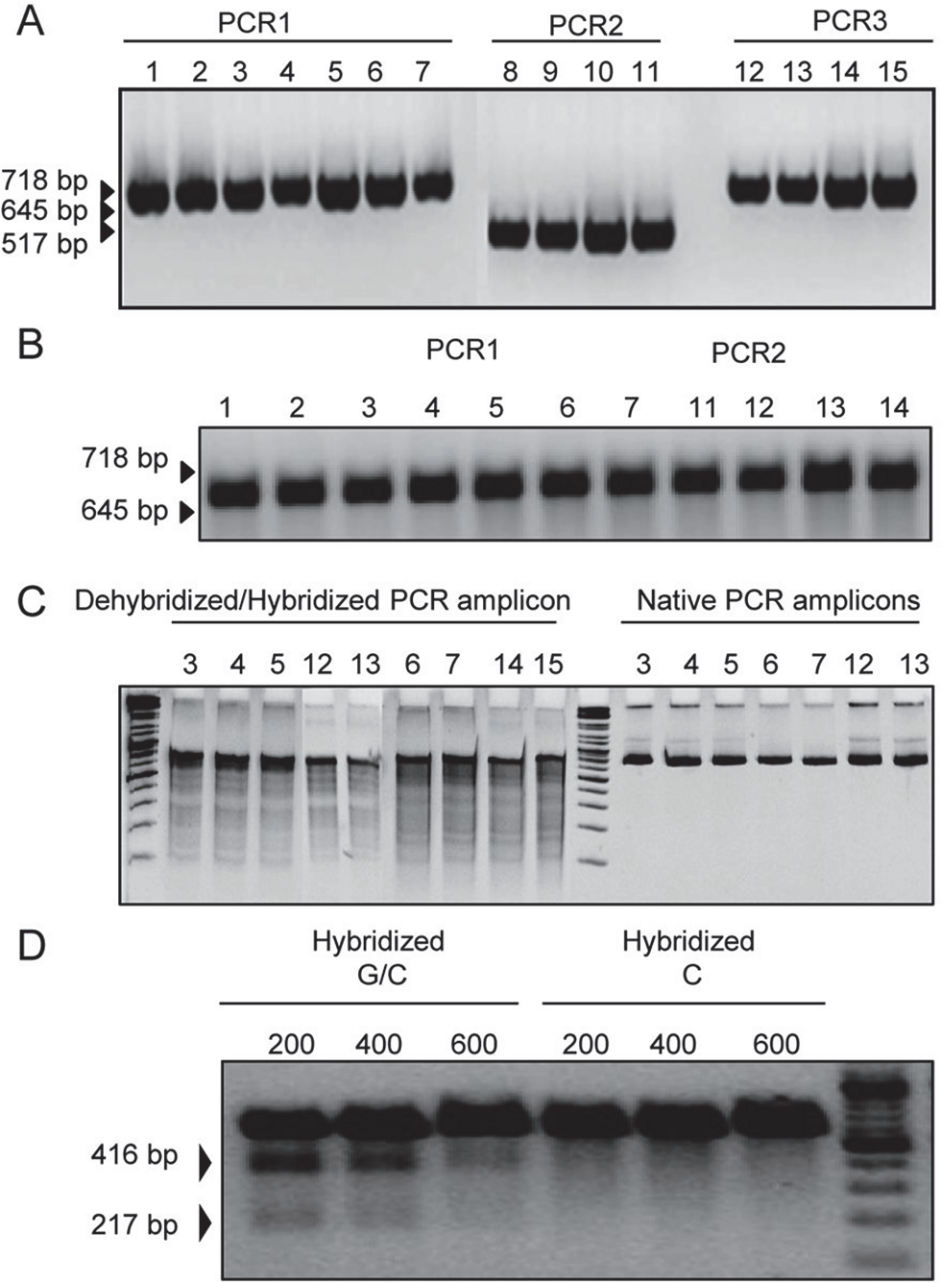
Results of the surveyor nuclease assay. **A.** PCR using genomic DNA as template for four different PCR (PCR1 = 645bp, PCR3 = 517 bp, PCR4 = 718 bp). After running on agarose gel, DNA concentration was estimated using ImageJ software. Lane 1–5, PK-15 cells transfected with 100 ng, 500 ng, 1 μg, 2 μg ZFN1/2 and 4 μg pLVTHM; lanes 8 and 12, PK-15 transfected with 2 μg ZFN1/2 each, lanes 9 and 13, PK-15 cells transfected with 4 μg pLVTHM. Lanes 6, 10 and 14 PERV-infected 293 cells transfected with 2 μg ZFN1/2 each and lanes 7, 11 and 15 with 4 μg pLVTHM. **B.** Agarose gel analysis of the rehybridisation of PCR amplicons shown in A. **C.** PAGE analysis of dehybridised and hybridised samples after incubation with 1 μl nuclease, 1 μl MgCl_2_ and 1 μl enhancer for 20 minutes. The sample numbers correspond to the samples described in A. **D.** Analysis of the G/C control of the surveyor nuclease assay. Three different amounts (200, 400, 600 ng) of DNA were treated with nuclease as described by the manufacturer and analyzed on an agarose gel.

In order to analyse whether the ZFN was functionally, usually a surveyor nuclease assay with DNA from ZFN treated cells was performed (see Materials and Methods). This assay recognizes mismatches in the DNA. After optimization and selection of an appropriate polymerase, PCR products were heated for dehybridisation and then cooled down slowly for re-annealing. At this stage the wild type sequences and mutated sequence if present will re-anneal building “bubbles” corresponding to the DNA mismatches, which can be then cut by the surveyor nuclease. All tested samples revealed the formation of many cleavage products, which can be seen as a multitude of bands (Fig 6). The band positions are reproducible for the same cell type independently from the reaction conditions and even when no ZFN plasmid was transfected. In order to test the nuclease for possible contamination by broad reacting nucleases, PCR products were treated directly with nuclease and no cleavage bands were detected (Fig 6B). When the G/C control was performed using two control plasmids with inserts that differ at a single base pair, cleavage of the heteroduplexes into two bands was observed (Fig 6D).

To confirm these results, the ZFN target sequences were amplified using the PCR1 primers (Table 2) and cloned using the Topo PCR cloning kit (Life Technologies, Frankfurt, Germany) as described by the manufacturer. The sequences of 24 cloned PERVs showed a high number of differences in the sequence of different proviruses (*Single Nucleotide Polymorphism*, SNPs) which were distributed all along the sequences (S4 Fig). These differences which could be targeted by the surveyor nuclease which leads to the high number of bands observed in the assay. This makes this assay not suitable for the analysis of the functionality of the ZFN in this system (Fig 6C).

**Table 2:** Primers used for the PCR.

ZFN Primers^a^		Sequences	Position
ZFN set 1 PCR 1	for	CGAAGGCACTACTGCTGGAA	3800..3819
	rev	CGTTGGTCATCCATCGGTCT	4444..4425
ZFN set 1 PCR 2	for	CAGGTGACCCTCCTCCAGTA	3727..3746
	rev	CGTTGGTCATCCATCGGTCT	4444..4425
ZFN set 1 PCR 3	for	CAGGTGACCCTCCTCCAGTA	3746..3765
	rev	CGTTGGTCATCCATCGGTCT	4262..4243

^a^Accession nr. AJ293656

## Discussion

Xenotransplantation using cells, tissues or organs from pigs may help to overcome the shortage of human transplants for the treatment of cell, tissue and organ failure. However, xenotransplantation may be associated with transmission of porcine microorganisms which may cause zoonoses (for review see [2, 6]). Most of the microorganisms will be eliminated by designated pathogen free breeding; however, PERVs are present in multiple copies in the genome of all pigs [5, 31] and therefore cannot be eliminated easily.

ZFNs allow gene editing in live cells by introducing a targeted DNA double-strand break at a specific gene locus. In most previous publications the knock-out of one or two genes in the genome was reported [18, 19, 22–30]. In contrast to this, there are more than 50 proviruses integrated in the genome of pigs [5, 31]. The presence of this high copy number was an extreme challenge for the application of ZFN. Since the PERVs are highly conserved in the pol region encoding for the reverse transcriptase (S1 Fig), the ZFN were designed to target this region.

A high expression of the ZFN in pig embryonic kidney cells (PK-15) and PERV-infected human embryonic cells (293) was observed and both proteins were found in the nucleus as shown by Western blot analysis, FRET and PLA, suggesting that they interact with the DNA. In addition, the results of the FRET analysis also suggest that both ZFN proteins act each with another.

ZFN induced cytotoxicity was previously observed in several cases and was thought to result most likely from cleavage at off-target sites [39, 40]. Analysis of the cleavage in in the yeast based assay demonstrated that the ZFNs are functional and since such sequences are not found in the pig genome (with exception of the multiple integrated proviruses), the cleavage should be specific. However, it was demonstrated previously that the cutting activity of ZFN pairs in yeast system does not guarantee their activity in mammalian systems [41].

The observed cytotoxicity is not due to the high amount of plasmid used for transfection. Cells transfected with the plasmids for both ZFN proteins are dying until day 5, whereas the cells transfected with the same amount of plasmid encoding only one ZFN protein, did not. This indicates that the amount of plasmid DNA was not toxic per se. Cytotoxicity was not observed using the same plasmids and uninfected 293 cells. It is compelling to suggest that due to the high number of proviral inserts the cellular genome was destabilised and the cells died. High cytotoxity was previously reported when ZFN were used, which were characterized by high off-target activity [39, 40, 42, 43]. But in our case off-target activity can be excluded because the ZFN were toxic only in PERV infected cells, not in the uninfected cells. Future studies could include plasmids with another promoter leading to a lower expression of the ZFN in the cells, or a repeated transfection of small amounts of plasmid allowing the cells to recover in between. We suggest that an exchange of the CMV promotor against a weaker or an inducible promotor such as the widely used tetracycline-inducible expression system could maintain the levels of protein expression [44]. The tet-based systems possibly may control ZFN-protein expression and reduce the cytoxicity during knock-out of PERV genes. A novel tetracycline inducible system was recently applied in double-transgenic pigs [45]. We propose that a significant lower expression of ZFN-proteins will facilitate the repairing of occurred double-strand DNA breaks. To be more precisely, the huge amount of PERV integrates must be cut by extremely low concentrations of ZFNs. This could be also achieved by transfecting ZFN mRNA instead of CMV driven plasmids. Finally, other strategies to knock-out PERVs may be performed such as using Transcription activator-like effector nucleases (TALEN) [46] or the clustered, regularly interspaced, short palindromic repeats (CRISPR)-associated protein (Cas) system (CRISPR/Cas9) [47].

## Conclusions

To increase the safety of xenotransplantation experiments were performed with the goal to knock out multiple proviral sequences of the PERV using the ZFN technology. Although the ZFN proteins were highly expressed in the nucleus and although both ZFN proteins were interacting, the expression of the ZFN induced an extreme cytotoxicity in the PERV-infected cells, but not in uninfected cells. Considering all these results, we came to the conclusion that the ZFN1 and ZFN2 were expressed and located in the nucleus. Despite the fact that the proviral target sequences of the ZFN were unique in comparison with the other cellular sequences, the observed cytotoxicity reminds that observed when ZNFs reacted off-target, possibly this is due to the high copy number of the PERV proviral copies in the genome of the porcine cells.

Furthermore, we showed that the surveyor nuclease assay, which is used to demonstrate the activity of the ZFN assay based on the mismatches between the mutated and unmutated sequences, cannot be used in the case of PERV proviruses which are characterized by multiple SNPs in the proviral sequences near the highly conserved target sequence. Modified experimental approaches or new strategies of gene editing should be used to eliminate as much as possible PERV sequences without destroying the genome.

## Supporting Information

S1 Fig Alignment of sequences inside the ZFN target region from different PERVs. The direct target sequences are in red. The name of the viruses, and their accession numbers are given.
(TIF)

S2 Fig Influence of ZFN on PERV expression. PK-15 or human 293 cells were untreated, or transfected with 2 or 5 μg of ZFN plasmid and PERV expression was analysed by real-time PCR using porcine cyclophilin or human GAPDH as control.
(TIF)

S3 Fig Expression of the ZFN fused to CFP and YFP in PK-15 cells. Different amounts (2 or 7.5 μg, respectively) of ZFN1 or ZFN 2 fused to CFP and YFP, were transfected into PK-15 cells and the expression of the ZFNs and the viability of the cells were analysed 1 and 5 days thereafter.
(TIF)

S4 Fig Alignment of the sequences from clones derived from the DNA of ZFN treated PK-15 cells. Genomic DNA from PK-15 cells was amplified by PCR using primers PCR1 (Materials and methods) and single clones were sequenced.
(DOCX)

## References

[1] Sachs DH, Sykes M, Robson SC, Cooper DK Xenotransplantation. Adv Immunol 2001;79: 129–223. 11680007 10.1016/s0065-2776(01)79004-9

[2] Fishman JA. Infection and xenotransplantation. Developing strategies to minimize risk. Ann N Y Acad Sci 1998;862: 52–66. 9928206 10.1111/j.1749-6632.1998.tb09117.x

[3] Patience C, Takeuchi Y, Weiss RA. Infection of human cells by an endogenous retrovirus of pigs. Nat Med 1997;3: 282–286. 9055854 10.1038/nm0397-282

[4] Wilson CA, Wong S, Muller J, Davidson CE, Rose TM, Burd P. Type C retrovirus released from porcine primary peripheral blood mononuclear cells infects human cells. J Virol 1998;72: 3082–3087. 9525633 10.1128/jvi.72.4.3082-3087.1998 PMC109758

[5] Takeuchi Y, Patience C, Magre S, Weiss RA, Banerjee PT, Le Tissier P et al Host range and interference studies of three classes of pig endogenous retrovirus. J Virol 1998;72: 9986–9991. 9811736 10.1128/jvi.72.12.9986-9991.1998 PMC110514

[6] Denner J, Tönjes RR. Infection barriers to successful xenotransplantation focusing on porcine endogenous retroviruses. Clin Microbiol Rev 2012;25: 318–343. 10.1128/CMR.05011-11 22491774 PMC3346299

[7] Denner J, Schuurman HJ, Patience C. The International Xenotransplantation Association consensus statement on conditions for undertaking clinical trials of porcine islet products in type 1 diabetes—chapter 5: Strategies to prevent transmission of porcine endogenous retroviruses. Xenotransplantation 2009;16: 239–248. 10.1111/j.1399-3089.2009.00544.x 19799764

[8] Denner J. Recombinant porcine endogenous retroviruses (PERV-A/C): A new risk for xenotransplantation? Arch Virol 2008;153: 1421–1426. 10.1007/s00705-008-0141-7 18584115

[9] Karlas A, Kurth R, Denner J. Inhibition of porcine endogenous retroviruses by RNA interference: Increasing the safety of xenotransplantation. Virology 2004;325: 18–23. 15231382 10.1016/j.virol.2004.04.022

[10] Ramsoondar J, Vaught T, Ball S, Mendicino M, Monahan J, Jobst P. Production of transgenic pigs that express porcine endogenous retrovirus small interfering RNAs. Xenotransplantation 2009;16: 164–180. 10.1111/j.1399-3089.2009.00525.x 19566656

[11] Dieckhoff B, Petersen B, Kues WA, Kurth R, Niemann H, Denner J. Knockdown of porcine endogenous retrovirus (PERV) expression by PERV-specific shRNA in transgenic pigs. Xenotransplantation 2008;15: 36–45. 10.1111/j.1399-3089.2008.00442.x 18333912

[12] Semaan M, Kaulitz D, Petersen B, Niemann H, Denner J. Long-term effects of PERV-specific RNA interference in transgenic pigs. Xenotransplantation 2012;19: 112–121. 10.1111/j.1399-3089.2012.00683.x 22497513

[13] Fiebig U, Stephan O, Kurth R, Denner J. Neutralizing antibodies against conserved domains of p15E of porcine endogenous retroviruses: Basis for a vaccine for xenotransplantation? Virology 2003;307: 406–413. 12667808 10.1016/s0042-6822(02)00140-x

[14] Kaulitz D, Fiebig U, Eschricht M, Wurzbacher C, Kurth R, Denner J. Generation of neutralising antibodies against porcine endogenous retroviruses (PERVs). Virology 2011;411: 78–86. 10.1016/j.virol.2010.12.032 21237477

[15] Waechter A, Denner J. Novel neutralising antibodies targeting the N-terminal helical region of the transmembrane envelope protein p15E of the porcine endogenous retrovirus (PERV). Immunol Res 2014;58: 9–19. 10.1007/s12026-013-8430-y 23729215

[16] Kim YG, Cha J, Chandrasegaran S. Hybrid restriction enzymes: Zinc finger fusions to Fok I cleavage domain. Proc Natl Acad Sci U S A 1996;93: 1156–1160. 8577732 10.1073/pnas.93.3.1156 PMC40048

[17] Smith J, Bibikova M, Whitby FG, Reddy AR, Chandrasegaran S, Carroll D. Requirements for double-strand cleavage by chimeric restriction enzymes with zinc finger DNA-recognition domains. Nucleic Acids Res 2000;28: 3361–3369. 10954606 10.1093/nar/28.17.3361 PMC110700

[18] Flisikowska T, Thorey IS, Offner S, Ros F, Lifke V, Zeitler B et al Efficient immunoglobulin gene disruption and targeted replacement in rabbit using zinc finger nucleases. PLoS One 2011;6: e21045 10.1371/journal.pone.0021045 21695153 PMC3113902

[19] Geurts AM, Cost GJ, Freyvert Y, Zeitler B, Miller JC, Choi VM et al Knockout rats via embryo microinjection of zinc-finger nucleases. Science 2009;325: 433 10.1126/science.1172447 19628861 PMC2831805

[20] Pruett-Miller SM, Connelly JP, Maeder ML, Joung JK, Porteus MH. Comparison of zinc finger nucleases for use in gene targeting in mammalian cells. Mol Ther 2008;16: 707–717. 10.1038/mt.2008.20 18334988

[21] Hurt JA, Thibodeau SA, Hirsh AS, Pabo CO, Joung JK. Highly specific zinc finger proteins obtained by directed domain shuffling and cell-based selection. Proc Natl Acad Sci U S A 2003;100: 12271–12276. 14527993 10.1073/pnas.2135381100 PMC218748

[22] Hauschild J, Petersen B, Santiago Y, Queisser AL, Carnwath JW, Lucas-Hahn A et al Efficient generation of a biallelic knockout in pigs using zinc-finger nucleases. Proc Natl Acad Sci U S A 2011;108: 12013–12017. 10.1073/pnas.1106422108 21730124 PMC3141985

[23] Lutz AJ, Li P, Estrada JL, Sidner RA, Chihara RK, Downey SM et al Double knockout pigs deficient in N-glycolylneuraminic acid and galactose alpha-1,3-galactose reduce the humoral barrier to xenotransplantation. Xenotransplantation 2013;20: 27–35. 10.1111/xen.12019 23384142

[24] Li P, Estrada JL, Burlak C, Tector AJ. Biallelic knockout of the alpha-1,3 galactosyltransferase gene in porcine liver-derived cells using zinc finger nucleases. J Surg Res 2013;181: e39–45. 10.1016/j.jss.2012.06.035 22795272

[25] Yang D, Yang H, Li W, Zhao B, Ouyang Z, Liu Z et al Generation of PPARgamma mono-allelic knockout pigs via zinc-finger nucleases and nuclear transfer cloning. Cell Res 2011;21: 979–982. 10.1038/cr.2011.70 21502977 PMC3203707

[26] Bao L, Chen H, Jong U, Rim C, Li W, Lin X et al Generation of GGTA1 biallelic knockout pigs via zinc-finger nucleases and somatic cell nuclear transfer. Sci China Life Sci 2014;57: 263–268. 10.1007/s11427-013-4601-2 24430555

[27] Lillico SG, Proudfoot C, Carlson DF, Stverakova D, Neil C, Blain C et al Live pigs produced from genome edited zygotes. Sci Rep 2013;3: 2847 10.1038/srep02847 24108318 PMC6505673

[28] Whyte JJ, Prather RS Cell Biology Symposium: Zinc finger nucleases to create custom-designed modifications in the swine (Sus scrofa) genome. J Anim Sci 2012;90: 1111–1117. 10.2527/jas.2011-4546 22038991

[29] Hauschild-Quintern J, Petersen B, Queisser AL, Lucas-Hahn A, Schwinzer R, Niemann H. Gender non-specific efficacy of ZFN mediated gene targeting in pigs. Transgenic Res 2013;22: 1–3. 10.1007/s11248-012-9647-6 22972477

[30] Watanabe M, Umeyama K, Matsunari H, Takayanagi S, Haruyama E, Nakano K et al Knockout of exogenous EGFP gene in porcine somatic cells using zinc-finger nucleases. Biochem Biophys Res Commun 2010;402: 14–18. 10.1016/j.bbrc.2010.09.092 20875794

[31] Le Tissier P, Stoye JP, Takeuchi Y, Patience C, Weiss RA. Two sets of human-tropic pig retrovirus. Nature 1997;389: 681–682. 9338777 10.1038/39489

[32] Owsianka AM, Patel AH. Hepatitis C virus core protein interacts with a human DEAD box protein DDX3. Virology 1999;257: 330–340. 10329544 10.1006/viro.1999.9659

[33] Chao CH, Chen CM, Cheng PL, Shih JW, Tsou AP, Lee YH. DDX3, a DEAD box RNA helicase with tumor growth-suppressive property and transcriptional regulation activity of the p21waf1/cip1 promoter, is a candidate tumor suppressor. Cancer Res 2006;66: 6579–6588. 16818630 10.1158/0008-5472.CAN-05-2415

[34] Olave IA, Reck-Peterson SL, Crabtree GR. Nuclear actin and actin-related proteins in chromatin remodeling. Annu Rev Biochem 2002;71: 755–781. 12045110 10.1146/annurev.biochem.71.110601.135507

[35] Pederson T, Aebi U. Actin in the nucleus: What form and what for? J Struct Biol 2002;140: 3–9. 12490148 10.1016/s1047-8477(02)00528-2

[36] Kremers GJ, Goedhart J, van Munster EB, Gadella TW Jr. Cyan and yellow super fluorescent proteins with improved brightness, protein folding, and FRET Forster radius. Biochemistry 2006;45: 6570–6580. 16716067 10.1021/bi0516273

[37] Soderberg O, Gullberg M, Jarvius M, Ridderstrale K, Leuchowius KJ, Jarvius J et al Direct observation of individual endogenous protein complexes in situ by proximity ligation. Nat Methods 2006;3: 995–1000. 17072308 10.1038/nmeth947

[38] Jarvius M, Paulsson J, Weibrecht I, Leuchowius KJ, Andersson AC, Wählby C et al In situ detection of phosphorylated platelet-derived growth factor receptor beta using a generalized proximity ligation method. Mol Cell Proteomics 2007;6: 1500–1509. 17565975 10.1074/mcp.M700166-MCP200

[39] Porteus MH, Baltimore D Chimeric nucleases stimulate gene targeting in human cells. Science 2003;300: 763 12730593 10.1126/science.1078395

[40] Alwin S, Gere MB, Guhl E, Effertz K, Barbas CF 3rd, Segal DJ et al Custom zinc-finger nucleases for use in human cells. Mol Ther 2005;12: 610–617. 16039907 10.1016/j.ymthe.2005.06.094

[41] Yang D, Yang H, Li W, Zhao B, Ouyang Z, Liu Z et al Generation of PPARr mono-allelic knockout pigs via zinc-finger nucleases and nuclear transfer cloning. Cell Res 2011;21: 979–982. 10.1038/cr.2011.70 21502977 PMC3203707

[42] Gabriel R, Lombardo A, Arens A, Miller JC, Genovese P, Kaeppel C et al An unbiased genome-wide analysis of zinc-finger nuclease specificity. Nat Biotechnol 2011;29: 816–823. 10.1038/nbt.1948 21822255

[43] Pattanayak V, Ramirez CL, Joung JK, Liu DR. Revealing off-target cleavage specificities of zinc-finger nucleases by in vitro selection. Nat Methods 2011;8: 765–770. 10.1038/nmeth.1670 21822273 PMC3164905

[44] Shaikh S, Nicholson LF. Optimization of the Tet-On system for inducible expression of RAGE. J Biomol Tech 2006;17: 283–292. 17028168 PMC2291795

[45] Kues WA, Schwinzer R, Wirth D, Verhoeyen E, Lemme E, Herrmann D et al Epigenetic silencing and tissue independent expression of a novel tetracycline inducible system in double-transgenic pigs. FASEB J 2006;20: 1200–1202. 16684801 10.1096/fj.05-5415fje

[46] Bogdanove AJ, Voytas DF. TAL effectors: Customizable proteins for DNA targeting. Science 2011;333: 1843–1846. 10.1126/science.1204094 21960622

[47] Shalem O, Sanjana NE, Hartenian E, Shi X, Scott DA, Mikkelsen TS et al Genome-scale CRISPR-Cas9 knockout screening in human cells. Science 2014;343: 84–87. 10.1126/science.1247005 24336571 PMC4089965

