# Supplementary figures and images for: Cytotoxic Effects during Knock Out of Multiple Porcine Endogenous Retrovirus (PERV) Sequences in the Pig Genome by Zinc Finger Nucleases (ZFN)
Source: PLoS One. 2015 Apr 24;10(4):e0122059. doi: 10.1371/journal.pone.0122059 (PMC4409370; doi:10.1371/journal.pone.0122059)

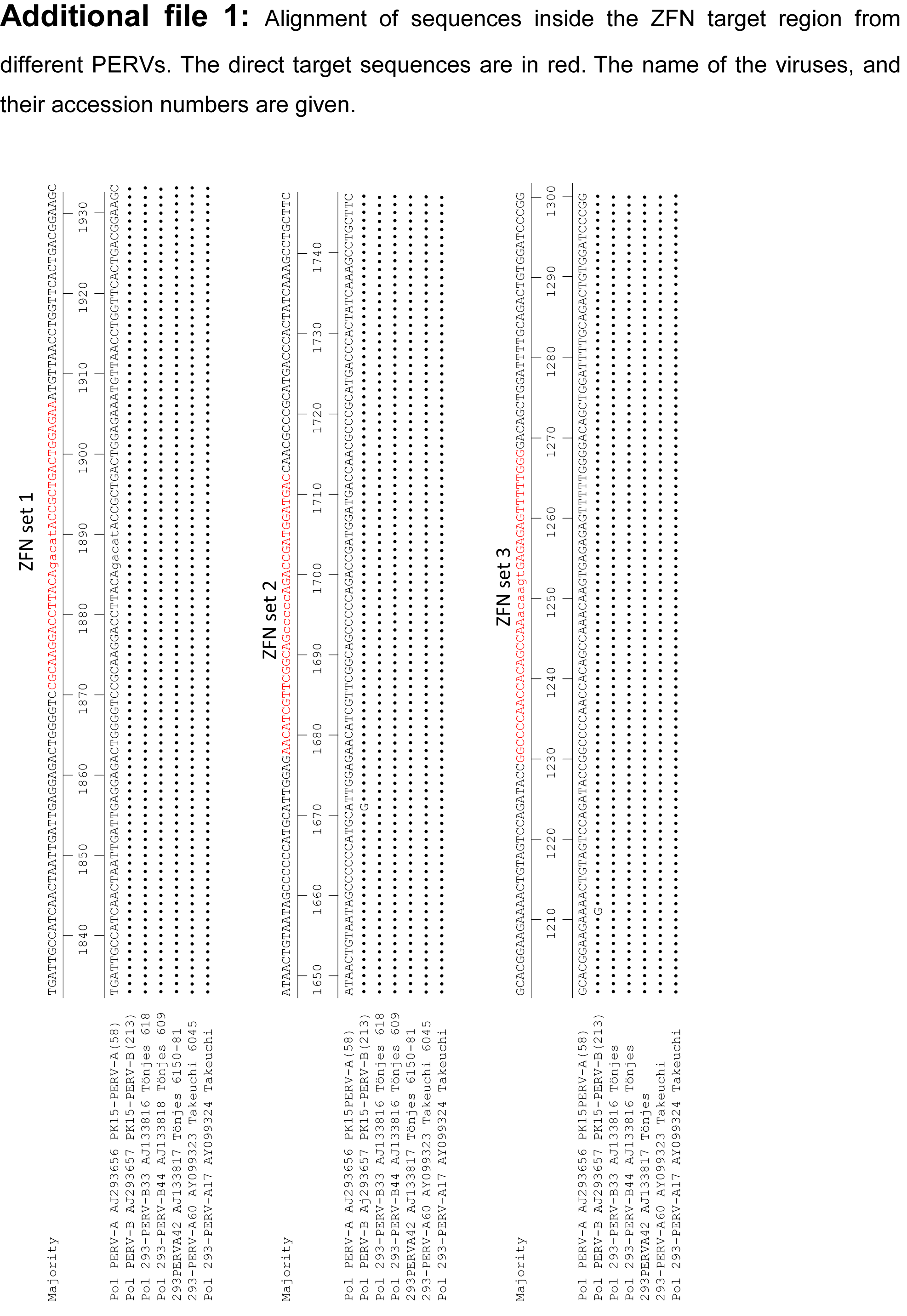

Supplement: S1 Fig — The direct target sequences are in red. The name of the viruses, and their accession numbers are given. (TIF) [file pone.0122059.s001.tif]

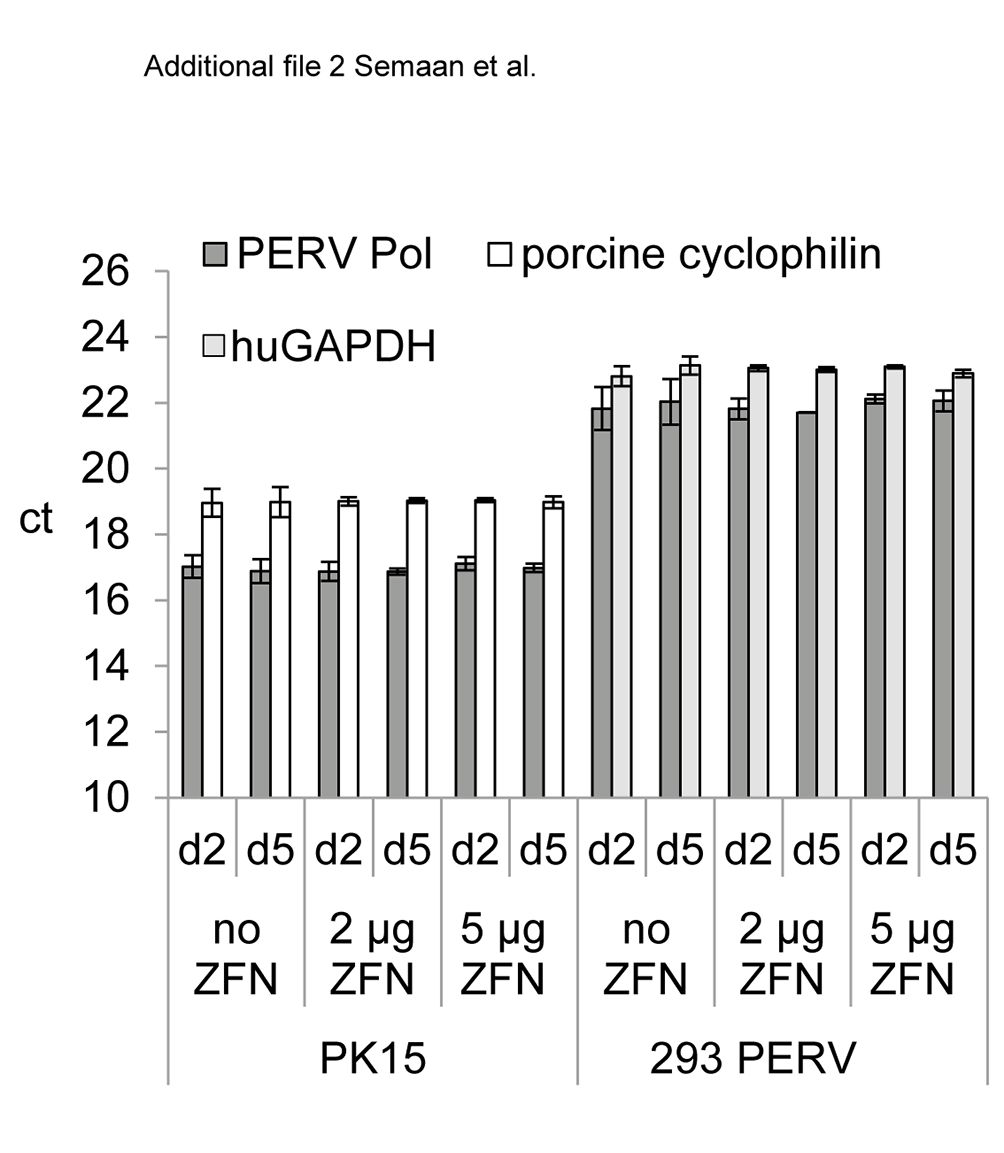

Supplement: S2 Fig — PK-15 or human 293 cells were untreated, or transfected with 2 or 5 μg of ZFN plasmid and PERV expression was analysed by real-time PCR using porcine cyclophilin or human GAPDH as control. (TIF) [file pone.0122059.s002.tif]

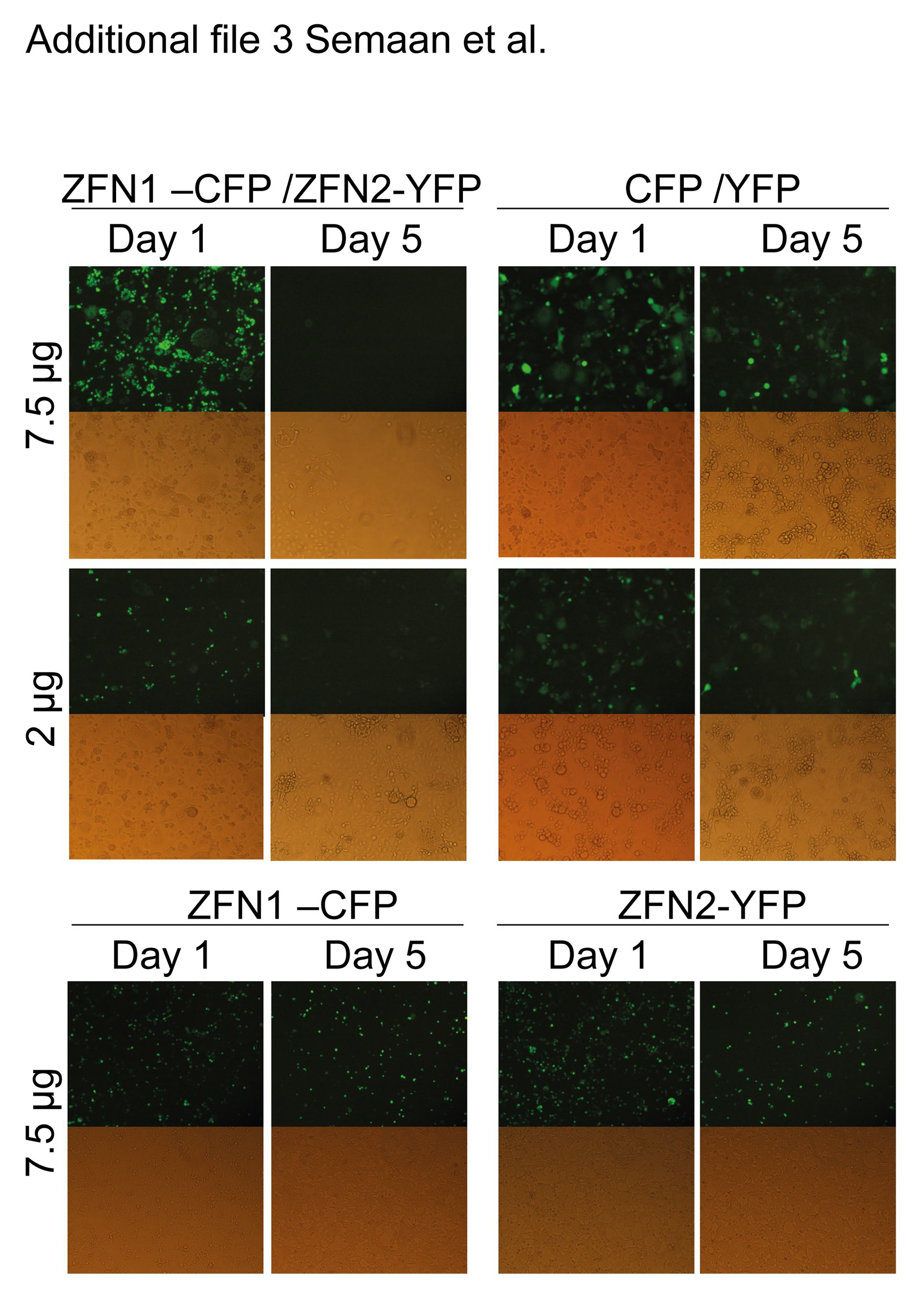

Supplement: S3 Fig — Different amounts (2 or 7.5 μg, respectively) of ZFN1 or ZFN 2 fused to CFP and YFP, were transfected into PK-15 cells and the expression of the ZFNs and the viability of the cells were analysed 1 and 5 days thereafter. (TIF) [file pone.0122059.s003.tif]
