# Supplementary material for: Cytotoxic Effects during Knock Out of Multiple Porcine Endogenous Retrovirus (PERV) Sequences in the Pig Genome by Zinc Finger Nucleases (ZFN)
Source: PLoS One. 2015 Apr 24;10(4):e0122059. doi: 10.1371/journal.pone.0122059 (PMC4409370; doi:10.1371/journal.pone.0122059)
Supplement: S4 Fig — Genomic DNA from PK-15 cells was amplified by PCR using primers PCR1 (Materials and methods) and single clones were sequenced. (DOCX) [file pone.0122059.s004.docx]

# Additional file 4: Alignment of the sequences from clones derived from the DNA of ZFN treated PK15 cells. Genomic DNA from PK15 cells was amplified by PCR using primers PCR1 (see Materials and methods) and single clones were sequenced.

**Majority CGTTGGTCATCCATCGGTCTGGGGGCTGCCGAACGATGTTCTCCAATGCATGGGGGGCTATTACAGTTATATTCTGTCCCAAAGTCAATTTGTCAGCGTC**

**---------+---------+---------+---------+---------+---------+---------+---------+---------+---------+**

**10 20 30 40 50 60 70 80 90 100**

**---------+---------+---------+---------+---------+---------+---------+---------+---------+---------+**

**revcomp 195-196 sequence CGTTGGTCATCCATCGGTCTGGGGGCTGCCGAACGATGTTCTCCAACGCATGGGGGGCTATTACAGTTATATTCTGTCCCAAAGTCAATTTGTCAGCGTC 100**

**revcomp MS_130926_01 .................................................................................................... 100**

**revcomp MS_130926_02 .................................................................................................... 100**

**revcomp MS_130926_03 ..............................................C..................................................... 100**

**revcomp MS_130926_04 ..............................................C..................................................... 100**

**revcomp MS_130926_05 .................................................................................................... 100**

**revcomp MS_130926_06 .................................................................................................... 100**

**revcomp MS_130926_07_20 .................................................................................................... 100**

**revcomp MS_130926_08_20 .................................................................................................... 100**

**revcomp MS_130926_09 .................................................................................................... 100**

**revcomp MS_130926_10 ...............................G...............................T.................................... 100**

**revcomp MS_130926_11 ..............................................C..................................................... 100**

**revcomp MS_130926_12_20 ..............................................C..................................................... 100**

**revcomp MS_130926_13_20 ..............................................C..................................................... 100**

**revcomp MS_130926_14 ...................A..........................C..................................................... 100**

**revcomp MS_130926_15 .................................................................................................... 100**

**revcomp MS_130926_16 ........................................T.....C..................................................... 100**

**revcomp MS_130926_17_20 .................................................................................................... 100**

**revcomp MS_130926_19 .........................T......................................C................................... 100**

**revcomp MS_130926_20 ..............-..CAA................................................................................ 100**

**revcomp MS_130926_21 .................................................................................................... 100**

**revcomp MS_130926_24_20 .................................................................................................... 100**

**Majority CTTGACCAGTATGGCCACAGCTGCGATAGCCTTCAGGCATACGGGCCAACCACTGGCTACAGGATCGAGCTTCTTTGACAGGTAGGCAACAGGTCTCCTC**

**---------+---------+---------+---------+---------+---------+---------+---------+---------+---------+**

**110 120 130 140 150 160 170 180 190 200**

**---------+---------+---------+---------+---------+---------+---------+---------+---------+---------+**

**revcomp 195-196 sequence CTTGACCAGTATGGCCACAGCTGCGATAGCCTTCAGGCATATGGGCCAACCACTGGCTACAGGATCGAGCTTCTTTGACAGGTAGGCAACAGGTCTCCTC 200**

**revcomp MS_130926_01 ..................................................................A................................. 200**

**revcomp MS_130926_02 ..................................................................A................................. 200**

**revcomp MS_130926_03 .................................................................................................... 200**

**revcomp MS_130926_04 .................................................................................................... 200**

**revcomp MS_130926_05 .................................................................A.................................. 200**

**revcomp MS_130926_06 .................................................................................................... 200**

**revcomp MS_130926_07_20 .................................................................A.................................. 200**

**revcomp MS_130926_08_20 ..................................................................A................................. 200**

**revcomp MS_130926_09 .................................................................................................... 200**

**revcomp MS_130926_10 ..................G................................................................................. 200**

**revcomp MS_130926_11 .................................................................................................... 200**

**revcomp MS_130926_12_20 .............................................A...................................................... 200**

**revcomp MS_130926_13_20 .................................................................................................... 200**

**revcomp MS_130926_14 .................................................................................................... 200**

**revcomp MS_130926_15 ..................................................................A................................. 200**

**revcomp MS_130926_16 .................G.......................T.......................................................... 200**

**revcomp MS_130926_17_20 ..................................................................A................................. 200**

**revcomp MS_130926_19 .........C........................................................A...............C................. 200**

**revcomp MS_130926_20 .................................................................A.................................. 199**

**revcomp MS_130926_21 ..................................................................A................................. 200**

**revcomp MS_130926_24_20 ..................................................................A................................. 200**

**Majority CATGGTCCTAGGGTTTGGGTTAAAACTCCCCGGGCTACTCCCTTACGCTCATCCACATAAAGGGTAAAGGGTTTAGTTACGTCAGGGAGGGCCAGAGCAG**

**---------+---------+---------+---------+---------+---------+---------+---------+---------+---------+**

**210 220 230 240 250 260 270 280 290 300**

**---------+---------+---------+---------+---------+---------+---------+---------+---------+---------+**

**revcomp 195-196 sequence CATGGTCCTAGAGTTTGGGTTAAAACTCCCCGGGCTACTCCCTTACGCTCATCCACATAAAGGGTAAAGGGTTTAGTCACGTCAGGGAGGGCCAGAGCAG 300**

**revcomp MS_130926_01 ....................................................................A..........A................... 300**

**revcomp MS_130926_02 .............................T...................................................................... 300**

**revcomp MS_130926_03 .............................................................................C...................... 300**

**revcomp MS_130926_04 ...........A.................................................................C...................... 300**

**revcomp MS_130926_05 .............................................G...................................................... 300**

**revcomp MS_130926_06 .................................................................................................... 300**

**revcomp MS_130926_07_20 .............................................G...................................................... 300**

**revcomp MS_130926_08_20 .............................T...................................................................... 300**

**revcomp MS_130926_09 .............................................................................C...................... 300**

**revcomp MS_130926_10 .............................................................................C...................... 300**

**revcomp MS_130926_11 ...........A.........................................T.......................C..A................... 300**

**revcomp MS_130926_12_20 .............................................T...................................................... 300**

**revcomp MS_130926_13_20 ...........A.................................................................C...................... 300**

**revcomp MS_130926_14 .............................................................................C...................... 300**

**revcomp MS_130926_15 .............................T...................................................................... 300**

**revcomp MS_130926_16 ......................G........................T................................A................... 300**

**revcomp MS_130926_17_20 .............................T...................................................................... 300**

**revcomp MS_130926_19 .............................T...................................................................... 300**

**revcomp MS_130926_20 .............................................G...................................................... 299**

**revcomp MS_130926_21 .............................T...................................................................... 300**

**revcomp MS_130926_24_20 .............................T...................................................................... 300**

**Majority GTGCGCTCAGCAGGGCCTTTTTGATAGCATCAAATGCCTTCTGGTGCTCAGGAGCCCAGGAGAATTCCCCTTTTTCTTTGGTTAGCGGGTAGAGTGGGGC**

**---------+---------+---------+---------+---------+---------+---------+---------+---------+---------+**

**310 320 330 340 350 360 370 380 390 400**

**---------+---------+---------+---------+---------+---------+---------+---------+---------+---------+**

**revcomp 195-196 sequence GTGCGCTCAGCAGGGCCTTTTTGATAGCATCAAATGCCTTCTGGTGCTCAGGAGCCCAGGAGAATTCCCCTTTTTCTTTGGTTAGTGGGTAGAGTGGGGC 400**

**revcomp MS_130926_01 .................................................................................................... 400**

**revcomp MS_130926_02 .................................................................................................... 400**

**revcomp MS_130926_03 .................................................................................................... 400**

**revcomp MS_130926_04 ..............................................T......................................T.............. 400**

**revcomp MS_130926_05 ..............................................................................................C..... 400**

**revcomp MS_130926_06 .................................................................................................... 400**

**revcomp MS_130926_07_20 .................................................................................................... 400**

**revcomp MS_130926_08_20 .................................................................................................... 400**

**revcomp MS_130926_09 .................................................................................................... 400**

**revcomp MS_130926_10 .....................................................................................T.............. 400**

**revcomp MS_130926_11 .....................................................................................T.............. 400**

**revcomp MS_130926_12_20 .................................................................................................... 400**

**revcomp MS_130926_13_20 .....................................................................................T.............. 400**

**revcomp MS_130926_14 .....................................................................T...............T.............. 400**

**revcomp MS_130926_15 ..............-..C...................................................................T.............. 399**

**revcomp MS_130926_16 .................................................................................................... 400**

**revcomp MS_130926_17_20 .................................................................................................... 400**

**revcomp MS_130926_19 .................................................................................................... 400**

**revcomp MS_130926_20 ..............................................................................................C..... 399**

**revcomp MS_130926_21 ........................................................-........................................... 399**

**revcomp MS_130926_24_20 .................................................................................................... 400**

**Majority TGCTAAGGTCGCAAACCCCGGGATCCACAGTCTGCAAAATCCAGCTGTCCCCAAAAACTCTCTCACTTGTTTGGCTGTGGTTGGGGCCGGTATCTGGACT**

**---------+---------+---------+---------+---------+---------+---------+---------+---------+---------+**

**410 420 430 440 450 460 470 480 490 500**

**---------+---------+---------+---------+---------+---------+---------+---------+---------+---------+**

**revcomp 195-196 sequence TGCTAAGGTCGCAAACCCCGGGATCCACAGTCTGCAAAATCCAGCTGTCCCCAAAAACTCTCTCACTTGTTTGGCTGTGGTTGGGGCCGGTATCTGGACT 500**

**revcomp MS_130926_01 .................................................................................................... 500**

**revcomp MS_130926_02 .................................................................................................... 500**

**revcomp MS_130926_03 .................................................................................................... 500**

**revcomp MS_130926_04 .................................................................................................... 500**

**revcomp MS_130926_05 ......................................................................C............................. 500**

**revcomp MS_130926_06 ..........................................................................................C......... 500**

**revcomp MS_130926_07_20 ..................T................................................................................. 500**

**revcomp MS_130926_08_20 .................................................................................................... 500**

**revcomp MS_130926_09 .................................................................................................... 500**

**revcomp MS_130926_10 .................................................................................................... 500**

**revcomp MS_130926_11 .................................................................................................... 500**

**revcomp MS_130926_12_20 .................................................................................................... 500**

**revcomp MS_130926_13_20 .................................................................................................... 500**

**revcomp MS_130926_14 .................G..................................C............................................... 500**

**revcomp MS_130926_15 .....T.............................................................................................. 499**

**revcomp MS_130926_16 ..T......T.......................................................................................... 500**

**revcomp MS_130926_17_20 .................................................................................................... 500**

**revcomp MS_130926_19 .................................................................................................... 500**

**revcomp MS_130926_20 .................................................................................................... 499**

**revcomp MS_130926_21 .................................................................................................... 499**

**revcomp MS_130926_24_20 .................................................................................................... 500**

**Majority ACAGTTTTCTTCCGTGCCTCCGTCAGCCATCGCTGCCCGCCCCGCAAACTGTACCCCAAGTATGTTACCTCTCTCCTGCAAATCTGGGCCTTCTTAGCGG**

**---------+---------+---------+---------+---------+---------+---------+---------+---------+---------+**

**510 520 530 540 550 560 570 580 590 600**

**---------+---------+---------+---------+---------+---------+---------+---------+---------+---------+**

**revcomp 195-196 sequence ACAGTTCTCTTCCGTGCCTCCGTCAGCCATCGCTGCCCGCCCCGCAAACTGTACCCCAAGTATGTTACCTCTCTCCTGCAAATCTGGGCCTTCTTAGCGG 600**

**revcomp MS_130926_01 ..................................................................................................A. 600**

**revcomp MS_130926_02 ...............................................G..................................................A. 600**

**revcomp MS_130926_03 .................................................................................................... 600**

**revcomp MS_130926_04 .T..........................................................................................T.....A. 600**

**revcomp MS_130926_05 ......C............................................................................................. 600**

**revcomp MS_130926_06 ......C.........................................................G................................... 600**

**revcomp MS_130926_07_20 .................................................................................................... 600**

**revcomp MS_130926_08_20 ..................................................................................................A. 600**

**revcomp MS_130926_09 ......................................A...T......................................................... 600**

**revcomp MS_130926_10 ......C...............................C............................................................. 600**

**revcomp MS_130926_11 ......C........................................C.................................................... 600**

**revcomp MS_130926_12_20 ..................................................................................................A. 600**

**revcomp MS_130926_13_20 ......C............................................................................................. 600**

**revcomp MS_130926_14 ......C............................................................................................. 600**

**revcomp MS_130926_15 ......C............................................................................................. 599**

**revcomp MS_130926_16 ....................T.................A..............T......................................T....T.. 600**

**revcomp MS_130926_17_20 ..................................................................................................A. 600**

**revcomp MS_130926_19 .................................................................................................... 600**

**revcomp MS_130926_20 ..................................................................................................A. 599**

**revcomp MS_130926_21 ..................................................................................................A. 599**

**revcomp MS_130926_24_20 ..................................................................................................A. 600**

**Majority AGGCTCTGTAGCCTAGGTCAGACAATTCCAGCAGTAGTGCCTTCGX**

**---------+---------+---------+---------+------**

**610 620 630 640**

**---------+---------+---------+---------+------**

**revcomp 195-196 sequence AGGCTCTGTAGCCTAGGTCAGACAATTCCAGCAGTAGTGCCTTCG 645**

**revcomp MS_130926_01 ............................................. 645**

**revcomp MS_130926_02 ............................................. 645**

**revcomp MS_130926_03 ........................-.....T..............A 645**

**revcomp MS_130926_04 ............................................. 645**

**revcomp MS_130926_05 ............................................. 645**

**revcomp MS_130926_06 ........................T.................... 645**

**revcomp MS_130926_07_20 ............................................. 645**

**revcomp MS_130926_08_20 ............................................. 645**

**revcomp MS_130926_09 ............................................. 645**

**revcomp MS_130926_10 ............................................. 645**

**revcomp MS_130926_11 ....................CC.CC.....-T.............A 645**

**revcomp MS_130926_12_20 ............................................. 645**

**revcomp MS_130926_13_20 ............................................. 645**

**revcomp MS_130926_14 ............................................. 645**

**revcomp MS_130926_15 .............................................A 645**

**revcomp MS_130926_16 ...............T............................. 645**

**revcomp MS_130926_17_20 ............................................. 645**

**revcomp MS_130926_19 ............................................. 645**

**revcomp MS_130926_20 .............................................A 645**

**revcomp MS_130926_21 .............................................A 645**

**revcomp MS_130926_24_20 ............................................. 645**
